# Bioactive compounds and antioxidant activity of wolfberry infusion

**DOI:** 10.1038/srep40605

**Published:** 2017-01-19

**Authors:** Yujing Sun, Japaer Rukeya, Wenyang Tao, Peilong Sun, Xingqian Ye

**Affiliations:** 1 Department of Food Science and Technology, Ocean college, Zhejiang University of Technology, Hangzhou 310014, China; 2 Department of Food Science and Nutrition, School of Biosystems Engineering and Food Science, Zhejiang University, Hangzhou 310058, China

## Abstract

An infusion of the wolfberry (*Lycium barbarum* L.) is a traditional Asian herbal tea. This is the most commonly consumed form of dried wolfberry worldwide, yet little scientific information on wolfberry infusions is available. We investigated the effects of making infusions with hot water on the color, the content of bioactive compounds (polysaccharides, polyphenols, flavonoids and carotenoids) and the antioxidant ability of wolfberry infusions. The contents of bioactive compounds and the antioxidant activity of a wolfberry infusion increased with increased infusion temperature and time. Total polysaccharides content (TPOC), total polyphenols (TPC), total flavonoids (TFC) and total carotenoids contents (TCC) were important for determining the antioxidant capacity of wolfberry infusions with the contribution to antioxidant activity in the order TPC > TFC > TCC > TPOC. Hierarchical cluster analysis indicated preparation conditions of 100 °C for 1~3 h, 90 °C for 2~3 h and 80 °C for 2.5~3 h were equivalent as regards the value of TPC, TPOC, TFC, TCC, FRAP, DPPH and ABTS. The results of this study suggest the length of time of making a wolfberry infusion in actual real life practice is too short and different dietary habits associated with the intake of wolfberry infusion might provide the same bioactive nutrients.

The wolfberry (*Lycium barbarum* L.) belongs to the Solanaceae. The genus *Lycium* comprises 87 recognized species and is distributed disjunctly in arid and subarid regions in temperate to subtropical zones in South America, North America, South Africa, Eurasia and Australia. China is currently the greatest supplier of wolfberry products in the world and commercial amounts of wolfberry are grown in the Chinese northwest regions: Ningxia, Inner Mongolia, Qinghai, Gansu, Shanxi and Hebei province. The wolfberry has been widely used as a traditional Chinese herb and functional food in China and other Asian countries including Vietnam, Korea, and Japan for more than 2500 years^1^,^2^. Recently, wolfberry has been marketed as foods and dietary supplements in many countries including North America, EU, Australia, New Zealand and Southeast Asia in various retail outlets, and consumers from Europe and America have increased the interest in wolfberries for their health and nutrient values^3^.

Wolfberries should be free of any insecticide and fungicide residue in order to reap the health benefits of wolfberry infusions. Wolfberry possess vital biological activities such as an anti-diabetic effect^4^, the prevention of age-related macular degeneration^5^, an immunomodulatory function^6^,^7^, anti-cancer^8^ and antioxidant activity^9^,^10^. Many components, including polysaccharides, flavonoids and carotenoids, which have been suggested to be associated with health-enhancing effects, have been found^11^,^12^,^13^,^14^. At the same time, a few negative functions were also reported. Lam *et al*.^15^ and Rivera *et al*.^16^ found the herbal-drug interaction between wolfberry and warfarin based on an increased international normalized ratio value. Gómez-Bernal *et al*.^17^ reported the systemic photosensitivity of goji berry.

Most studies have focused on their bioactive compounds such as polysaccharide and pharmacological activities in the literatures. Beside its medical use, dried wolfberry is also a popular ingredient in Chinese domestic cuisine, they are consumed in tonic soups in combination with vegetables, rice congee (porridge) and chicken or pork. The berries are also drunk as a tea, wine, they are usually boiled as a herbal tea (alone or with chrysanthemum flowers (*Chrysanthemum morifolium* or *Chrysanthemum indicum*), red jujubes (*Zizisiphus jujube*)) or with tea (*Camellia sinensis*) and soaked in liquor to make wolfberry wine. An infusion of the dried berries as a herbal tea is the most commonly consumed form of wolfberry in China. However, there are few scientific evidences regarding anything of long traditional uses as food including the wolfberry infusion until now, only Li *et al*.^18^ mentioned the wolfberry infusion during the investigation the antioxidant capacities and total phenolic contents of 223 medicinal plants infusion at a settled condition (100 °C, 30 min). In this study wolfberry was soaked in hot water in the form of powder which is different with the real wolfberry infusion in the form of a whole fruit, the studied infusion conditions and bioactive compounds are limited. In fact, Dietary habits (culinary processes) might have a significant effect on the composition and biological activity of a wolfberry infusion, including the vessel (cup or pot) in which it is prepared, how much water is used and its temperature, how long it is left to infuse and whether it is dunked or stirred and left in or removed before consumption.

In this study, we sought to investigate the factors that actually influence bioactive compounds and biological activities in the domestic preparation of wolfberry infusions, thus providing information directly useful to the consumer. This approach is in contrast to that generally adopted, where the conditions established for studies tend to be laboratory specific. The primary purpose of this report was to provide the most comprehensive assessment to date of the amounts of bioactive compounds and antioxidant activity per 150 mL cup of wolfberry infusion.

## Results and Discussion

### Effects of infusion temperature, length of time and number of infusions on the color of wolfberry infusion

**Figure 1:**
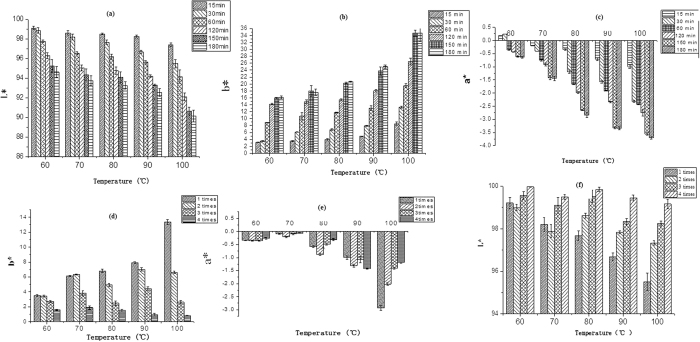
The L*, b*, a* value of wolfberry infusions prepared at different temperatures for different lengths of time (**a**), (**b**), (**c**), and for different infusion times (**d**), (**e**), (**f**).

The color of the wolfberry infusions was described by L*, a* and b*. L* represents the lightness of the color (L* = 0 yields black and L* = 100 indicates diffuse white; specular white may be higher), a* represent its position between red/magenta and green (a*, negative values indicate green while positive values indicate magenta), and b* represents its position between yellow and blue (b*, negative values indicate blue and positive values indicate yellow). The variation of color of wolfberry infusions as influenced by temperature, length of time and the number of infusion cycles are summarized in Fig. 1.

The L* value of wolfberry infusions decreased with increased temperature and time of infusion and increased with increased infusion time indicating the infusion had a lighter color at low temperature or short brewing time or multiple infusion times (Fig. 1(a)–(d)). The b* value increased with increased infusion temperature and time and decreased with increased infusion times (Fig. 1(b)–(e)). The a* value decreased with the increase of temperature, time and multiple infusion times (Fig. 1(c)–(f)). The variations of L*, a*, b* at different conditions were connected with the difference of polysaccharides, polyphenols, carotenoids contents in wolfberry infusion, respectively.

### Effects of infusion temperature, length of time and number of infusions on the total polysaccharides content (TPOC) of wolfberry infusion

**Figure 2:**
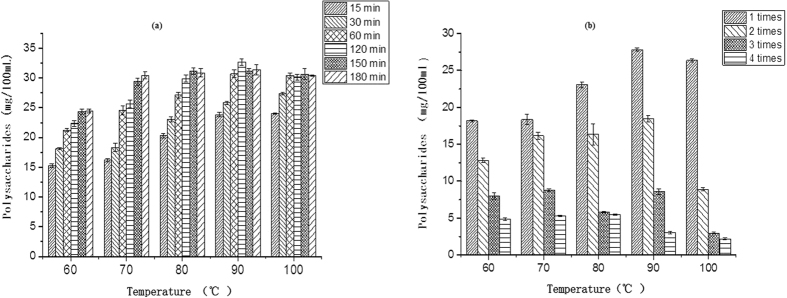
Polysaccharides content of wolfberry infusion at different temperatures for different lengths of time (**a**), and for different infusion times (**b**).

Figure 2 shows the variation of TPOC of wolfberry infusion with infusion temperature, time and number of infusions. TPOC increased with temperature when infusion time increased from 0–30 min, and the TPOC first increased then decreased with temperature when infusion time increased from 60–180 min with a peak value at 90 °C (Fig. 2(a)). The TPOC decreased with increased infusion time, and most of the wolfberry polysaccharides were dissolved in the infusion during the first and second infusion (Fig. 2(b)).

### Effects of infusion temperature, length of time and number of infusions on the total carotenoids content (TCC) of wolfberry infusion

**Figure 3:**
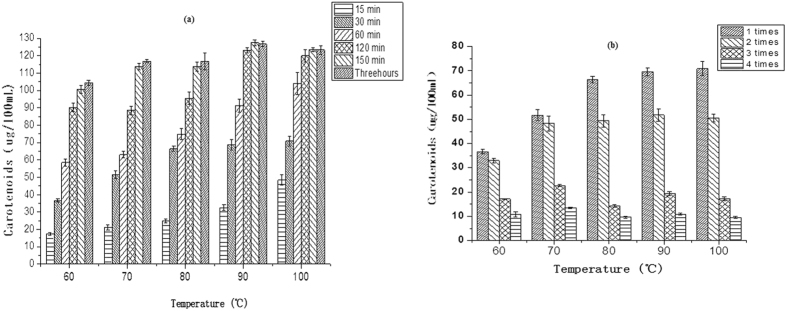
Carotenoids content of wolfberry infusion at different temperatures for different lengths of time (**a**), and for different infusion times (**b**).

TCC of wolfberry infusion was low (18–125 μg/100 mL) because of the poor solubility of carotenoids in water. TCC of wolfberry infusion increased with temperature when the infusion time increased from 0–60 min and first increased then decreased with temperature when infusion time increased from 120–180 min with a peak value at 90 °C (Fig. 3(a)). These results indicated wolfberry carotenoids were degraded when the infusion time was >60 min at 100 °C. TCC decreased with increased infusion time and was significantly higher in the first and second infusion compared to the third and fourth.

### Effects of infusion temperature, length of time and number of infusions on the total phenolics content (TPC) and total flavonoids content (TFC) of wolfberry infusion

**Figure 4:**
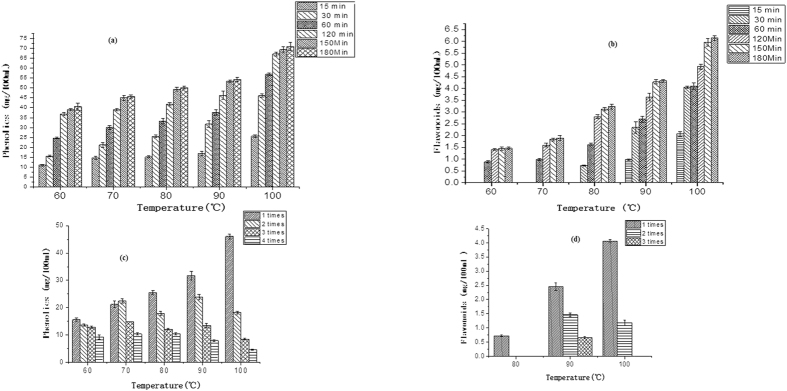
Total phenolics, total flavonoids contents of wolfberry infusion at different temperatures for different lengths of time (**a**), (**b**) and for different infusion times (**c**), (**d**).

The effects of infusion temperature, time and number of infusions on TPC and TFC of a wolfberry infusion are shown in Fig. 4. TPC and TFC increased with increased temperature and time, and TPC and TFC of an infusion at 100 °C was significantly higher compared to other temperatures tested. At 60 °C and 70 °C, TFC was not detected at 15 min infusion time, indicating that the flavonoids dissolve slowly at these temperatures. At 80 °C, TFC was detected, but only in the first infusion. TPC in a wolfberry infusion was higher (7.5–21.2 mg/g) than the results for wolfberry powder infusion at 100 °C, 30 min reported by Li *et al*.^18^ (6.38 mg/g), but lower compared to a black tea infusion (range 22.5–311.0 mg/g)^19^.

### Effects of infusion temperature, time and number of infusions on antioxidant activity (AA) of wolfberry infusions

**Figure 5:**
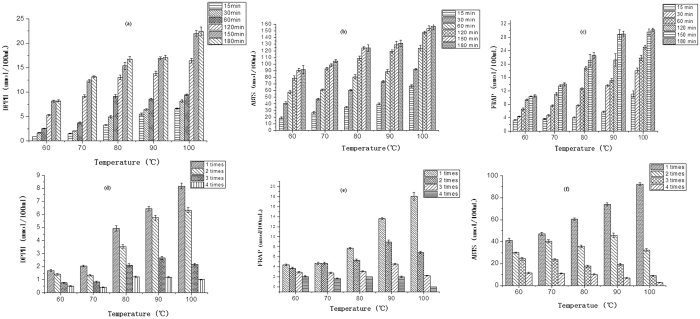
ABTS, DPPH, FRAP of a wolfberry infusion at different temperatures for different lengths of time (**a**), (**b**) (**c**) and for different infusion times (**d**), (**e**), (**f**). The error bars show ± S.D.

The effects of infusion temperature, length of time and number of infusions on the antioxidant activity of wolfberry infusion were investigated. The antioxidant activity of wolfberry infusion was evaluated by FRAP, ABTS and DPPH assays. The antioxidant activity (AA) of a wolfberry infusion increased with increased temperature (AA at 100 °C was about threefold higher compared to 60 °C), increased with increased time from 0–150 min and was constant at 150 min (Fig. 5(a)–(c)). Figure 5(d)–(f) indicates the AA of wolfberry infusion decreased with length of infusion time at decreased temperature, and the differences of AA were significant at higher temperature compared to lower temperature. We concluded most of the AA of a wolfberry can be made available by infusion at 90 °C or at 100 °C, but the wolfberry has to be infused several times at temperatures <70 °C to obtain the same yield. The trend of variation of AA was related positive to the variation of phytochemicals under different conditions. Correlation coefficients of TPC, TPOC, TFC, TCC, FRAP, DPPH and ABTS are given in Table 1; TPC, TPOC, TFC and TCC are correlated significantly (*p* < 0.01) with FRAP, DPPH and ABTS. These results indicate TPC, TPOC, TFC and TCC have major roles in the antioxidant capacity of wolfberry infusion. The general trend of contribution to AA between the phytochemicals was in the order TPC > TFC > TCC > TPOC in accord with to their correlation coefficients with AA.

The contents of polysaccharides, carotenoids, polyphenols and AA in wolfberry infusion increased slightly with increased infusion temperature and length of time. The higher temperature might disrupt the berry cells, causing more phytochemicals to be released readily into the water. Other workers reported similar results. Xu^20^ found an aqueous extract of Satsuma mandarin (*Citrus unshui*) peel at 100 °C had higher TPC and AA compared to the lower temperatures. Samaniego-Sánchez^21^ found an infusion of green tea (*Camellia sinensis*) at 90 °C for 1 min had higher AA compared to 80 °C or 70 °C.

### Hierarchical cluster analysis

**Figure 6:**
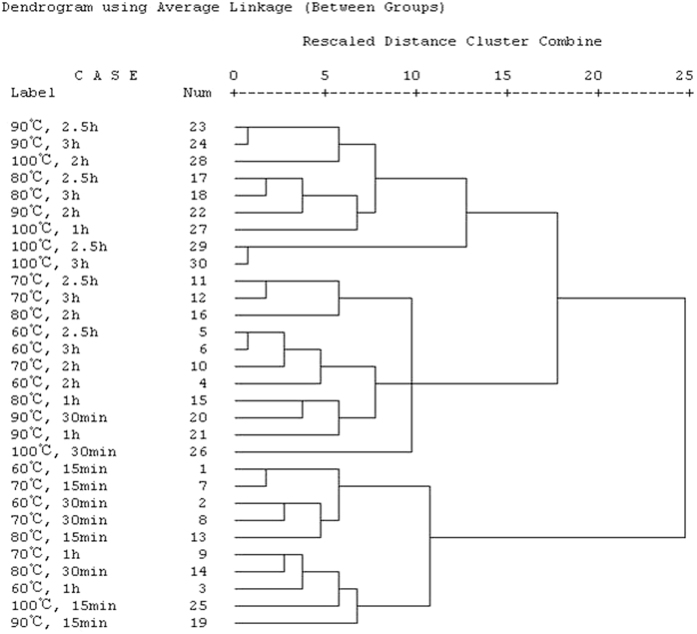
Dendogram obtained after hierarchical cluster analysis for wolfberry infusion under all conditions studied here.

In an attempt to determine equivalent infusion conditions, hierarchical cluster analysis (HCA) was used on the value of TPC, TPOC, TFC, TCC, FRAP, DPPH and ABTS obtained in this study. In HCA, the similarity between samples is established using the method of between groups linkage and Euclidean distance. Figure 6 shows the dendogram obtained from HCA in this study. It is apparent that three clusters repeat throughout the series: one has preparation conditions of 100 °C for 1~3 h, 90 °C for 2~3 h and 80 °C for 2.5~3 h. The second cluster has preparation conditions of 100 °C for 30 min, 90 °C for 30 min~1 h, 80 °C for 1~2 h, 70 °C for 2~3 h and 60 °C for 2~3 h. The third has the conditions of 100 °C for 15 min, 90 °C for 15 min, 80 °C for 15~30 min, 60 °C for 15 min~1 h and 70 °C for 15 min~1 h. The value of three clusters decreased successively from up to down of the dendrogram and the preparation conditions in the same cluster are equivalent on TPC, TPOC TFC, TCC, FRAP, DPPH and ABTS of the samples analyzed. These results indicated different dietary intakes of wolfberry infusion might provide people with the same bioactive compounds.

## Conclusion

We investigated the influence of factors close to reality on bioactive compounds and antioxidant activity during preparation of wolfberry infusions, thus providing information that may be useful to the consumer directly. Effects of dietary habits on color, bioactive compounds and the antioxidant ability of wolfberry infusions were investigated. The total contents of TPC, TPOC, TFC and TCC as well as AA in wolfberry infusions were increased with infusion temperature and length of time, which were important for the antioxidant capacity of wolfberry infusions. The contribution to AA was in the order TPC > TFC > TCC > TPOC. HCA indicated preparation conditions of 100 °C for 1~3 h, 90 °C for 2~3 h and 80 °C for 2.5~3 h were equivalent as regards TPC, TPOC, TFC, TCC, FRAP, DPPH and ABTS values. This study shows the length of time of making a wolfberry infusion in actual real life practice is too short and the different dietary intakes of wolfberry infusion might provide people with the same bioactive compounds.

## Methods

### Chemicals

Chlorogenic acid, Trolox (6-hydroxy-2,5,7,8-tetramethylchroman-2-carboxylic acid), gallic acid and rutin were purchased from Sigma Aldrich Co. (St. Louis, MO, USA). 2,4,6-Tri-2-pyridyl-s-triazin (TPTZ), 2,2-diphenyl-1-picrylhydrazyl radical (DPPH), 2,2-azinobis-(3-ethylbenzothiazoline-6-sulfonic acid) (ABTS), Folin–Ciocalteu phenol reagent and other reagents were analytical grade purchased from Aladdin Reagent Database Inc. (Shanghai, China).

### Materials

Dried wolfberries (*L. barbarum* L.) were a kind gift from Golmud Yilin Wolfberry Technology Development Co., Ltd., Golmud City, Qinghai Province. The moisture content was 17.86% (w/w).

### Preparation of wolfberry infusions

Fresh water (150 mL) was added to a flask containing 5 g of dried wolfberries, which were infused a different number of times for different lengths of time at different temperatures. The infusions were filtered by passage through a 45 μm pore size PTFE disposable syringe filter and kept at a constant volume of 150 mL.

### Measurement of total polysaccharides content (TPOC)

Total polysaccharides were measured by the phenol–sulfuric acid method using glucose as the standard^22^.

### Preparation of the standard solution

A 50 mg sample of glucose (dried at 105 °C) was dissolved in distilled water, transferred to a 50 mL volumetric flask, made to a final volume of 50 mL with water and mixed well. The content of glucose is 1 mg/mL. Samples (0.15, 0.3, 0.45, 0.6, 0.75 and 0.9 mL) of the standard glucose solution (1 mg/mL) were placed into a 10 mL volumetric flasks, made to a final volume of 10 mL with distilled water and mixed well to produce standard glucose solutions with concentrations of glucose at 15, 30, 45, 60, 75 and 90 g/mL, respectively.

### Preparation of the calibration curve

A 1 mL sample of 5% (w/v) phenol was added to 1 mL of standard glucose solution, mixed well and then 5 mL of concentrated sulfuric acid solution was added rapidly. After 5 min at room temperature, the mixture was heated in a water bath for 15 min, then cooled rapidly to room temperature. The optimal absorption wavelength was 490 nm (*A*_490_). Distilled water was used as the blank.

### Determination of the conversion factor

A 1 mL sample of filtered wolfberry infusion was placed into a 10 mL volumetric flask, made to 10 mL with distilled water and mixed well. A 1 mL sample of the diluted solution was used and *A*_490_ was measured according to the method of the standard curve using distilled water as the blank. The concentration and content of glucose in the wolfberry polysaccharide infusion was calculated according to the regression equation. The conversion factor was calculated as:


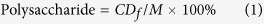


where *C* is the concentration of glucose in the sample, *D* is the dilution, *f* is the conversion factor and *M* is the mass of dried wolfberry.

### Determination of total carotenoids content (TCC)

TCC of the wolfberry infusion was analyzed by the Lambert–Beer law^23^,^24^.


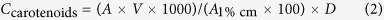


where C_carotenoids_ is the contents of total carotenoids, *A* is the absorbance value, *V* is the infusion volume, *A*_(1% cm)_ is the theoretical absorbance value of 1 g/L solute at 1 cm optical pathlength, and D is dilution factor. The carotenoids content of wolfberry is expressed as zeaxanthin equivalents, and A (1% cm) of zeaxanthin is 2480.

### Colorimetric analysis of wolfberry infusion

The color of the wolfberry infusion was monitored with an SC-80C fully automatic colorimeter (Beijing Kangguang Instrument Co. Ltd., China).

### Total phenolics content (TPC) and total flavonoids content (TFC) analysis

A 0.2 mL sample of a wolfberry infusion was added to a 25 mL colorimetric cylinder containing 0.5 mL of Folin–Ciocalteu reagent, shaken thoroughly and left for 5 min. Then 5 mL of 5% (w/v) Na_2_CO_3_ was added, the solution was vortex mixed and made to 25 mL with distilled water. After 90 min, *A*_750_ was measured in a UV-2550 spectrophotometer (Shimadzu Co, Kyoto, Japan) using distilled water as a blank. A calibration curve was prepared using a standard solution of gallic acid and the results for TPC were expressed as mg gallic acid equivalents per 100 mL of infusion^25^,^26^.

TFC was measured by the following method. Briefly, 1 mL of wolfberry infusion was placed into a 10 mL volumetric flask containing 4.0 mL of distilled water and stirred well. Next, 5% NaNO2 (w/v) was added and mixed. After 5 min, 0.3 mL of 10% Al(NO3)3 was added and mixed. After another 5 min, 4 mL of 1 M NaOH solution was added and mixed before the volume was made to 10 mL with distilled water. *A*_510_ was measured after 10 min at room temperature and the results were expressed in mg rutin equivalents per 100 mL of fresh sample weight according to a calibration curve constructed using rutin as standard solution.

### Determination of antioxidant activity (AA)

AA was evaluated by the ferric reducing antioxidant power (FRAP), 2, 2-diphenyl-1-picrylhydrazyl radical (DPPH) and 2, 29-azinobis-(3-ethylbenzothiazoline-6-sulfonic acid) (ABTS) assays.

### Ferric reducing antioxidant power (FRAP) assay

Fresh FRAP reagent was prepared by mixing 0.1 M acetate buffer (pH 3.6), 10 mM TPTZ dissolved in 40 M HCL and 20 mM ferric chloride dissolved in distilled water (10:1:1, by vol.). A 0.2 mL sample of wolfberry infusion was added to 4.9 mL of FRAP reagent. After 15 min, *A*_593_ was measured with a Shimadzu UV-visible 2550 spectrophotometer and Trolox solution was used to construct the calibration curves. The results are expressed as Trolox equivalent antioxidant capacity (TEAC) mg/g sample dry weight (DW)^27^.

### DPPH free radical-scavenging assay

A 0.2 mL sample of wolfberry infusion was added to 3.9 mL of 0.1 mM DPPH dissovled in methanol then kept in darkness for 30 min at room temperature. *A*_517_ was measured with a Shimadzu UV-visible 2550 spectrophotometer. Trolox solution was used to construct the calibration curves and the results are expressed as TEAC mg/g DW^28^.

### ABTS free radical-scavenging assay

Potassium persulfate dissolved in distilled water (140 mM, 88 μL) was added to 5 mL of 7 mM ABTS dissolved by H_2_O_2_, kept in darkness at 25 °C for 12–16 h, then diluted with ethanol to *A*_734_ of 0.70 ± 0.02. ABTS cation solution (7 mM 3.9 mL) was added to 0.2 mL of wolfberry infusion, mixed thoroughly then kept at 25 °C for 106 min. *A*_734_ was measured with a Shimadzu UV-2550 spectrophotometer. A control (0.1 mL of 80% (v/v) ethanol, 3.9 mL of ABTS solution) was prepared and a calibration curve was constructed for the absorbance reduction and concentration of the Trolox standard. The ABTS radical-scavenging ability is expressed as TEAC mg/g DW^28^.

### Statistical analysis

All samples were prepared and analyzed in triplicate and the results are presented as mean ± standard deviation. One-way analysis of variance was used to determine the significance of any test. Statistically significant difference between means was determined by least significant difference. The Pearson correlation coefficient (*R*) and *P*-value were used to express correlations and their significance. *p* ≤ 0.01 was adopted as the criterion for statistically significant difference (SPSS for Windows, Release 15.0, SPSS Inc.).

## Additional Information

**How to cite this article:** Sun, Y. *et al*. Bioactive compounds and antioxidant activity of wolfberry infusion. *Sci. Rep.* **7**, 40605; doi: 10.1038/srep40605 (2017).

**Publisher's note:** Springer Nature remains neutral with regard to jurisdictional claims in published maps and institutional affiliations.

## Figures and Tables

**Table 1:** Correlation coefficients of TPC, TPOC, TFC, TCC, FRAP, DPPH and ABTS.

	TPC	ABTS	FRAP	DPPH	TPOC	TFC
ABTS	0.988**					
FRAP	0.952**	0.957**				
DPPH	0.936**	0.944**	0.957**			
TPOC	0.865**	0.903**	0.827**	0.800**		
TFC	0.940**	0.927**	0.972**	0.929**	0.776**	
TCC	0.948**	0.964**	0.898**	0.906**	0.897**	0.842**

^**^Correlation was set as statistically significant at *p* ≤ 0.01 (two-tailed distribution).
